# A case‐comparison study of pregnant women with mitochondrial disease – what to expect?

**DOI:** 10.1111/1471-0528.15667

**Published:** 2019-03-27

**Authors:** CL Feeney, AZ Lim, E Fagan, A Blain, A Bright, J Maddison, H Devine, J Stewart, RW Taylor, GS Gorman, DM Turnbull, V Nesbitt, R McFarland

**Affiliations:** ^1^ NHS Specialised Service for Rare Mitochondrial Disorders of Adults and Children Newcastle‐upon‐Tyne UK; ^2^ Wellcome Centre for Mitochondrial Research Institute of Neuroscience Newcastle University Newcastle‐upon‐Tyne UK; ^3^ MRC Centre for Neuromuscular Diseases Institute of Neurology University College London London UK; ^4^ Newcastle Fertility Centre International Centre for Life Newcastle UK

**Keywords:** Birthweight, breathing difficulties, gestation, gestational diabetes, hypertension, m.3243A>G, mitochondria, mitochondrial disease, *MTTL1*, pregnancy, pregnant women with mitochondrial disease

## Abstract

**Objective:**

Mitochondrial disease is a disorder of energy metabolism that affects 1 in 4300 adults in the UK. Pregnancy is associated with physiological demands that have implications for energy metabolism. We were interested to know how pregnancy was affected in women with mitochondrial disease, particularly those with the most common pathogenic mutation m.3243A>G.

**Design:**

Retrospective case‐comparison study.

**Population/Setting:**

Sixty‐seven women with genetically confirmed mitochondrial disease from the UK Mitochondrial Diseases Cohort and 69 unaffected women participated.

**Methods:**

Participants answered questionnaires regarding each of their pregnancies. Patients were divided into two groups according to genetic mutation, with those harbouring m.3243A>G comprising a single group.

**Main outcome measures:**

Pregnancy‐related complications, mode of delivery, gestational age and birthweight of newborns.

**Results:**

Of 139 live births in the comparison group, 62 were in the m.3243A>G group and 87 were in the ‘all other mutations’ group. Pregnancies of women with the m.3243A>G mutation had significantly more gestational diabetes (odds ratio [OR] = 8.2, 95% CI 1.3–50.1), breathing difficulties (OR = 7.8, 95% CI 1.0–59.1) and hypertension (OR = 8.2, 95% CI 3.1–21.5) than the comparison group. Only half of the pregnancies in the m.3243A>G group had normal vaginal delivery, with emergency caesarean section accounting for 24.2% of deliveries. Babies were born significantly earlier to mothers harbouring m.3243A>G with 53.3% of them preterm (<37 weeks). These babies were also more likely to require resuscitation and admission.

**Conclusion:**

Women who carried the m.3243A>G mutation appeared to be at higher risk of complications during pregnancies, caesarean section and preterm delivery than the unaffected women or those with other forms of mitochondrial disease.

**Tweetable abstract:**

Pregnant women with mitochondrial disease – m.3243A>G mutation – are at greatly increased risk of complications and preterm delivery.

## Tweetable abstract

Pregnant women with mitochondrial disease – m.3243A>G mutation – are at greatly increased risk of complications and preterm delivery.

## Introduction

### Mitochondrial disease

Mitochondrial disease resulting from nuclear and mitochondrial DNA genetic mutations collectively affects 1 in 4300 adults in the UK.^1^, ^2^ The most common pathogenic point mutation of the mitochondrial genome is the m.3243A>G in the *MTTL1* gene, with a carrier rate of 1 in 400 individuals.^3^, ^4^, ^5^ This maternally inherited mutation is implicated in several clinical syndromes such as mitochondrial encephalopathy, lactic acidosis and stroke‐like episodes, maternally inherited deafness and diabetes, and progressive external ophthalmoplegia.^6^, ^7^ Affected patients may also exhibit non‐syndromic features such as seizures, myopathy, ptosis, migraine, ataxia, gut dysmotility or cognitive decline.^7^ Some case reports have highlighted patients with mitochondrial disease who have suffered obstetric complications or presented during pregnancy with deterioration in their health.^8^, ^9^ Carriers of m.3243A>G seemed to have higher rates of obstetric complications than the general population.^10^ However, it remains unclear if other pathogenic mutations are also associated with obstetric complications.

### Background

Mitochondrial disease typically affects organs with high energy requirements such as the brain, skeletal muscle, heart and liver.^2^ The normal physiological adaptations of pregnancy are likely to have bio‐energetic consequences that increase the need for mitochondrial ATP production. These additional demands on ATP supply during pregnancy in the context of mitochondrial insufficiency may be an important mechanism in maternal and fetal complications.

### Study objective

We hypothesised that mitochondrial dysfunction, with its attendant decrease in ATP production, would predispose women with mitochondrial diseases to high‐risk pregnancy and delivery, even before their disease became clinically apparent. We attempted to identify these risks by interviewing women who had been diagnosed with mitochondrial disease. In the UK, there has been no large‐scale study into the outcomes of pregnant women with mitochondrial disease. Knowledge gained from this study may provide insight into understanding the role of mitochondrial genetic defects in adverse pregnancy outcomes.

## Methods

### Patient and comparison groups

Women aged >16 years with pathogenic genetic mutations alongside compatible clinical and biochemical features were recruited from the UK MRC Mitochondrial Disease Cohort into the patient group. This group was further classified into two genotypic subgroups; those with m.3243A>G mutation and those with all other types of genetic mutations causing mitochondrial disease. Genetic mutations were analysed and confirmed by the NHS Highly Specialised Service for Rare Mitochondrial Disorders diagnostic laboratory in Newcastle University, UK. In the comparison group, we used unaffected family members of patients to minimise socio‐economic status and ethnicity as confounding factors. An additional convenience sample of female staff, regardless of roles, in three hospital wards in the Royal Victoria Infirmary Newcastle were also recruited to increase the size of the comparison group. Women in the comparison group were matched to patients by their age in the same year.

### Main outcome measures

A patient focus group was consulted to design the questionnaire, together with input from the specialist mitochondrial clinical team. Four main outcome measures were selected for this study:

1. *Fetal loss*: miscarriage – spontaneous loss of pregnancy before 24 weeks of gestation; stillbirth – baby delivered with no signs of life at or after 24 weeks of gestation
2. *Pregnancy‐related complications*: gestational diabetes mellitus (GDM); hypertension; breathing difficulties (feeling of excessive breathlessness during pregnancy); anaemia; and antepartum vaginal bleeding
3. *Mode of delivery*: normal vaginal delivery; assisted vaginal delivery with forceps or vacuum; emergency and elective caesarean section
4. *Newborn outcomes*: gestational age; birthweight; preterm birth (<37 completed weeks of gestation); very preterm birth (<32 completed weeks of gestation); birth centiles – using customised centile calculations from NHS Gestation Network;^11^ admission to Special Care Baby Unit (SCBU); neonatal resuscitation.

These measures were self‐reported so cannot be precisely defined. They are the individual impressions of the respondents. The questionnaire was not piloted nor validated but feedback on clarity and feasibility was provided by a small group of five patients.

### Data collection

All potential recruits received information regarding the proposed study, together with an explanatory letter, consent form and prepaid envelope. After consenting to the study, a research nurse (CF) telephoned all participants to arrange a face‐to‐face or telephone interview. Our structured questionnaire entailed asking women to recall outcomes for each of their individual pregnancies and deliveries. To quantify the outcomes of this study, questions, asked in plain English, required ‘Yes’, ‘No’ or numerical responses from the participants. Pregnancy‐related complications, delivery outcomes and newborn outcomes were what these women had experienced or what had been communicated to them by their respective clinical team (obstetricians/midwives). Technical medical terminology in the questionnaire was made easy for participants to understand. Answering the questionnaire took approximately 20 minutes. Any omissions or ambiguities were clarified by follow‐up telephone conversations with the research nurse.

### Statistics

All identifying details were anonymised and statistical analyses were conducted using SPSS (v23). For the purposes of statistical comparisons, participants were grouped into three groups, namely m.3243A>G, other mutations and comparison groups. Presence of pregnancy‐related complications, mode of delivery, birth resuscitation requirement and admissions to SCBU were dichotomised (yes/no) for analysis, except for gestation and birthweights, of which absolute numbers were recorded. Data were expressed as frequencies or means ± standard deviation (SD) if normally distributed. Binominal data of each outcome measure from these three groups underwent chi‐square 3 × 2 analysis (including Fisher's exact test for counts below five), and parametric data had one‐way analysis of variance (anova) to look for any differences. A *P*‐value < 0.05 (adjusted for multiple testing using the Benjamini and Hochberg method, where appropriate) was considered statistically significant. If statistical analysis revealed any significant differences, groups were then compared with each other in pairs using chi‐square test or post‐hoc Tukey's test. Generalised linear models were used to test the relationship between genetic mutation and a variety of outcome measures, with the individual unique maternal anonymised identifier included as a random effect in all models to account for multiple births per woman. Akaike Information Criteria were used to assess the relative model fit.

### Patient involvement

The idea for this research was first conceived following anecdotal reports of pregnancy‐related complications and preterm deliveries raised by women at our Highly Specialised NHS Service for Rare Mitochondrial Disorder clinic in Newcastle in 2013. Our questionnaire had also undergone scrutiny by patient focus groups and had subsequently been enhanced by outcome measures that mattered to patients. Results from this study had been made available to patients in our Reproductive Advice clinics for mitochondrial disease.

### Ethics and funding

This study obtained institutional ethical approval from Sunderland NRES Committee (Ref 11/NE/0028) and complied with the Declaration of Helsinki. This work received support from the Wellcome Centre for Mitochondrial Research (DT, RM, AL), Biomedical Research Council UK (GSG) and the Medical Research Council UK (VN, HD, DT and RM).

## Results

### Basic demographics of women

**Figure 1:**
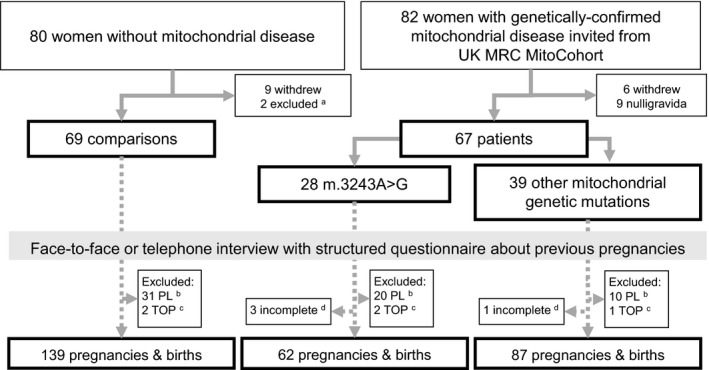
Participation flow chart. ^a^Two women excluded because of twin pregnancies. ^b^PL, pregnancy loss or miscarriage; ^c^TOP, termination of pregnancy. ^d^Incomplete for analysis – no reliable pregnancy details disclosed on stillbirths.

Eighty‐two women with genetically confirmed mitochondrial disease agreed to participate; of whom six withdrew and nine did not fulfil the inclusion criteria (nulligravida). Of these 67 women who completed the questionnaire, 28 had the m.3243A>G mutation, 5 had the m.8344A>G mutation, 8 had the large‐scale single deletions of mitochondrial DNA (mtDNA), 11 had the other mtDNA mutations (m.15669G>A, m.5650A>G, m.14709T>C, m.9176T>C, m.1053A>G, m.7587T>C, m.4267A>G) and 15 had nuclear genetic mutations (*PEO1*,* TWNK*,* POLG*,* RRM2B*,* ETFDH* multiple deletions of mtDNA). None of the women who were approached to be contemporaneous comparisons declined initially. Although 80 of these women agreed to participate, nine subsequently withdrew. Two women who had twin pregnancies were omitted from matching with the patient group, which only had singleton pregnancies. Consequently, there were 69 women in the comparison group, 28 recruits with m.3243A>G mutations and 39 recruits with other mitochondrial genetic mutations to be studied (Figure 1). Only one of the women with m.3243A>G was diagnosed at the birth of their first child (from family pedigree tracing) and none of the women with m.3243A>G considered themselves to have any mitochondrial disease manifestations at the time of their pregnancies.

The age of respondents at the time of pregnancy was not statistically different between the m.3243A>G patient group (27.1 years, SD = 4.7, 95% CI 25.9–28.3), the other mutations patient group (27.3 years, SD = 6.5, 95% CI 26.0–28.7) and the comparison group (27.8 years, SD = 4.9, 95% CI 27.0–28.7) as determined by one‐way anova (*F *=* *16.5, *P *=* *1.00) despite the initial withdrawals from the study (Table 1). Women in the other mutations group (55.7 years, SD = 10.4, 95% CI 52.4–59.1) were older at the time of interview than their comparisons (47.8 years, SD = 12.0, 95% CI 44.9–50.7) and m.3243A>G mutation (47.0 years, SD = 11.3, 95% CI 42.6–51.4). At the time of recruitment, all three groups had similar mean weight (*F *=* *2.37, *P *=* *0.343) and body mass index (*F *=* *0.542, *P *=* *0.1.00). Although there were more smokers in the patient groups (*n* = 10) than in the comparison group (*n* = 4), these differences did not reach statistical significance (*P* = 0.345). After accounting for multiple births, there was no difference between all three groups in the proportion of women reporting irregular periods or a history of miscarriages (Table 1). No significant difference was observed in exercise tolerance between patient and comparison groups before pregnancy.

**Table 1:** Summary of basic demographics of women in this study

	m.3243A>G	Other mutations	Comparison	*P*‐value
Number of women eligible	28	39	69	
Mean age at delivery (years)	27.1 (4.7, 26–28)	26.0 (6.5, 16–47)	27.8 (4.9, 27–29)	0.609
Mean weight (kg)	60.9 (15.1, 40–107)	66.1 (13.7, 44–114)	67.9 (14.3, 44–114)	0.343
Mean body mass index (kg/m^2^)	24.2 (6.0, 18–46)	25.2 (5.8, 18–44)	25.4 (4.7, 20–48)	0.609
Mean age at interview (years)	47.0 (11.3, 26–72)	55.7 (10.4, 32–72)	47.8 (12.0, 22–76)	<0.001
Women with history of smoking	4 (14.3%)	6 (15.4%)	4 (5.8%)	0.345
Women reporting regular periods	25 (89.3%)	37 (94.9%)	57 (82.6%)	0.345
Women with history of miscarriage	11 (39.3%)	9 (23.1%)	18 (26.1%)	0.437
Number of miscarriages	20	10	31	0.309
Number of terminations of pregnancy	2	1	2	0.364
Number of incomplete data	3	1	0	0.309

One‐way anova used to compare differences of age at delivery, weight, body mass index and age at interview among the three groups. Results expressed as mean followed by (SD, 95% CI). Categorical or nominal data expressed in absolute numbers followed by (%) and compared between all three groups using chi‐square or Fisher's exact test. *P*‐value < 0.05 was deemed statistically significant among three groups, after adjustment for multiple testing

### Fetal loss

There were 87 reported pregnancies in the m.3243A>G group, 99 pregnancies in the other mutations group and 172 pregnancies in the comparison group. The number of pregnancies per respondent in all three groups did not differ significantly (*P* = 0.495). Women with m.3243A>G reported 20 miscarriages while the other mutations and comparison groups reported 10 and 31 miscarriages, respectively. There were no significant differences in the history of miscarriages among all three groups (*P* = 0.309). Five women (three patients and two unaffected women) had terminations; one patient and one unaffected woman declined to provide reasons, while the other three women terminated for medical reasons; trisomy 18 (unaffected woman), spina bifida (patient) and chorionic villus biopsy (for m.3243A>G patient who had been diagnosed from family pedigree before pregnancy). All four of the recorded stillbirths occurred in two mitochondrial disease patients (other mutations group, *n* = 1; m.3243A>G, *n* = 3). There were insufficient details about their pregnancies with these stillbirths for analysis of pregnancy‐related complications and birth.

### Pregnancy‐related complications

**Figure 2:**
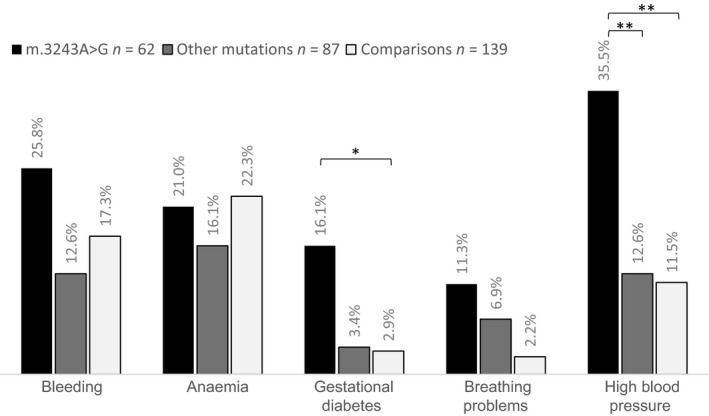
Percentage of pregnancies in which women had experienced bleeding, anaemia, gestational diabetes, breathing problems and high blood pressure. Women with m.3243A>G experienced significantly more (gestational) diabetes and high blood pressure during pregnancies than women in comparison groups *P <* *0.05; **P ≤* *0.001. (Generalised linear models, accounting for multiple births).

After accounting for pregnancy losses and incomplete data, the number of pregnancies available for review was 62 in the m.3243A>G, 87 in the other mutations and 139 in the contemporaneous comparison groups (Table 2). Comparison between these groups after accounting for multiple births showed significant differences in three outcome measures during pregnancy, namely GDM (*P *=* *0.008), problems with breathing (*P* = 0.03) and hypertension (*P* < 0.001). GDM was reported in 10 of the 62 (16.1%) m.3243A>G pregnancies, compared with only 3.4% in the other mutations and 2.9% in the comparison groups. Women with m.3243A>G (11.3%) experienced more breathing difficulties during pregnancy than the other two groups. More than a third of women in the m.3243A>G (35.5%) group had hypertension whereas only 6.9% and 2.2% had hypertension in the other mutations and comparison groups, respectively (Figure 2). Odds of having GDM (OR 8.2, 95% CI 1.3–50.1), breathing difficulties (OR 7.8, 95% CI 1.0–59.1) and hypertension (OR 8.2, 95% CI 3.1–21.5) were higher in pregnancies of mothers with m.3243A>G than those in the unaffected group. There were no statistical differences in bleeding (*P *=* *0.144) and anaemia (*P *=* *0.531) during pregnancies between all three groups. When complications were reviewed together, again after accounting for multiple births per mother, women with m3243A>G had significantly more complications as a whole than women in the other two groups (*P* = 0.006). There were significantly more complications for first pregnancies when compared with subsequent pregnancies (*P* = 0.015).

**Table 2:** Summary of outcomes for all pregnancies analysed in the study

	m.3243A>G	Other mutations	Comparison	*P*‐value
Number of pregnancies (%)	62 (100)	87 (100)	139 (100)	0.495
**Problems during pregnancy**				
*During your pregnancy, did you experience any of the following complications?*				
Bleeding (%)	16 (25.8)	11 (12.6)	24 (17.3)	0.144
Anaemia (%)	13 (21.0)	14 (16.1)	31 (22.3)	0.531
Diabetes of pregnancy (%)	10 (16.1)	3 (3.4)	4 (2.9)	**0.005**
Problems with breathing (%)	7 (11.3)	6 (6.9)	3 (2.2)	**0.030**
High blood pressure (%)	22 (35.5)	11 (12.6)	16 (11.5)	**<0.001**
**Mode of delivery**				
Did you have?				
Normal vaginal delivery (%)	31 (50.0)	58 (66.7)	102 (73.4)	**0.010**
Forceps or vacuum delivery (%)	12 (19.4)	13 (14.9)	21 (15.1)	0.709
Elective caesarean section (%)	4 (6.5)	7 (8.0)	6 (4.3)	0.619
Emergency caesarean section (%)	15 (24.2)	9 (10.3)	10 (7.2)	**0.008**
**Newborn outcome**				
Mean gestation (days) (95% CI)	253 (245–261)	277 (273–280)	279 (277–281)	**<0.001**
Mean birth weight (g) (95% CI)	2779 (2529‐3029)	3313 (3170‐3456)	3429 (3314–3545)	**<0.001**
Median birth centiles	58.5th	51st	58th	0.329
Number of babies < 37 weeks (%)	33 (53.2)	8 (9.2)	10 (7.3)	**<0.001**
Number of babies < 32 weeks (%)	8 (12.9)	2 (2.3)	1 (0.7)	**<0.001**
*Did your baby require resuscitation (at birth)?*				
Yes (%)	13 (21.0)	7 (8.0)	8 (5.8)	**0.004**
*Did your baby require admission to special care?*				
Yes (%)	33 (53.2)	11 (12.6)	22 (15.8)	**<0.001**

Bold values denote *P* < 0.05.

Categorical or nominal data expressed in absolute numbers followed by (%) and compared between all three groups using chi‐square or Fisher's exact test. One‐way anova used to compare differences of gestation days and birthweight among the three groups. Results expressed as mean followed by (SD, 95% CI). *P*‐value < 0.05 was deemed statistically significant among three groups, after adjustment for multiple testing

### Mode of delivery

**Figure 3:**
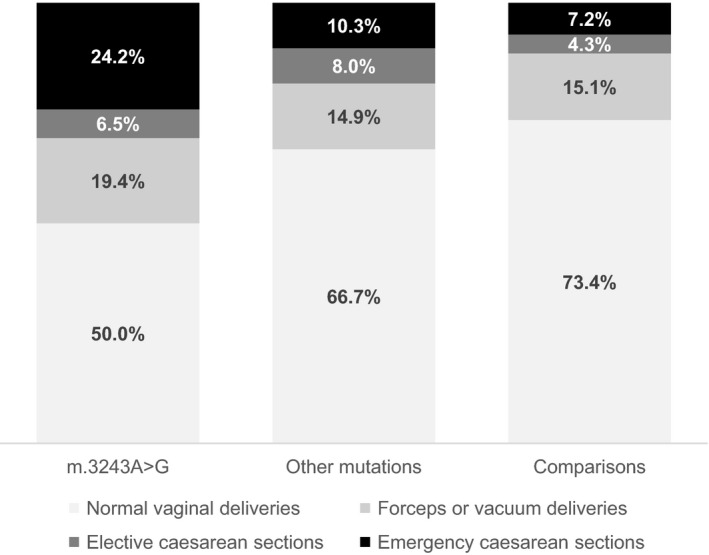
Modes of delivery of three study groups. Only half of the deliveries in the m.3243A>G group were normal vaginal deliveries; significantly fewer than in the comparison group. In the remaining half of the m.3243A>G deliveries, emergency caesarean section was the most common mode of delivery.

Only 50% of the pregnancies of mothers with m.3243A>G mutations were delivered vaginally without any interventions, in contrast to 66.7% in the other mutations group and 73.4% in the comparison group (*P *=* *0.01) (Figure 3). Emergency caesarean section was the second most likely mode of delivery after normal vaginal delivery for carriers of m.3243A>G mutations (24.2%). Both other mutations (10.3%) and comparison (7.2%) groups had significantly lower emergency caesarean section rates (*P *=* *0.008). Hence, pregnancies of women with m.3243A>G had much higher odds compared with unaffected women (OR 7.6, 95% CI 2.3–36.1) to undergo emergency caesarean sections, after accounting for multiple births per woman. Other modes of delivery, including forceps, vacuum delivery, and elective caesarean section, showed no statistical difference between all three groups.

### Newborn outcomes

**Figure 4:**
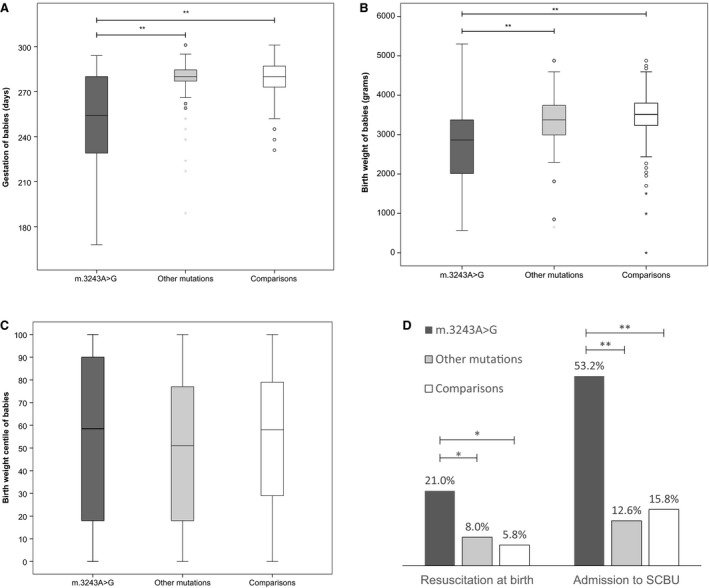
Newborn outcomes. (A) Gestation of babies (in days); (B) Birthweight of babies (in grams). Babies born to mothers with m.3243A>G had significantly lower gestational age and birthweight than both other mutations and comparison groups. (C) Birthweight centile corrected for gestation. No difference between all three groups. (D) Babies born to mothers with m.3243A>G had significantly more resuscitations at birth and admissions to SCBU. *P <* *0.05; **P ≤* *0.001 (Generalised linear models, accounting for multiple births).

More than half (53.3%) of the 62 live births to women with m.3243A>G were preterm, or <37 weeks. Compared with the comparison group (7.3%) and the other mutations group (9.2%), these differences were statistically significant (*P *<* *0.001). There were also significantly more very preterm babies delivered at <32 weeks (12.9%) to mothers with m.3243A>G than the comparison group (0.7%) and the other mutations (2.3%) group (*P* < 0.001). Mothers with m.3243A>G had a much higher risk of having very preterm babies (OR = 7.6, 95% CI 1.6–36.1) and or preterm babies (OR = 41.2, 95% CI 3.3–507.3) than unaffected women. At birth, about one in five babies of m.3243A>G mothers required resuscitation. Number of admissions to SCBU of these babies was significantly higher than for babies born to the other two groups (*P *<* *0.001) (Figure 4). Babies born to mothers with the m.3243A>G mutation had more than six times the risk of requiring resuscitation at birth (OR = 6.7, 95% CI 1.9–23.7) and 11 times the risk of admission to SCBU (OR = 11.2, 95% CI 3.5–36.1) compared with babies born to unaffected mothers in the comparison group.

Comparison of gestational age (*F* = 38.7, *P *<* *0.001) and birthweight (*F* = 16.2, *P *<* *0.001) of babies born to mothers in all three groups showed significant differences. Post‐hoc comparisons using the Tukey honestly significant difference test demonstrated that the mean gestational age (in days) and birthweight (in grams) for the m.3243A>G group were both significantly different from those reported in the comparison and other mutations group (*P *<* *0.001) Once multiple births were accounted for, m.3243A>G births occurred on average 26 days earlier than in the comparison group. Gestational age at birth in the other mutations group did not significantly differ from those in the comparison group. Given that the babies born early were likely to have lower birthweight, a customised birth centile was plotted for each child. Comparisons of birth centiles among all three groups did not show a significant difference (*F *=* *1.12, *P *=* *0.329) (Figure 4). Taken together, these observations suggested that babies of mothers with m.3243A>G were born significantly earlier, but not with lower birthweights after correcting for gestation (i.e. there was no increase in smallness for gestational age).

## Discussion

### Main findings

Our study highlighted that pregnancies of women who had m.3243A>G, the most common genetic mutation for adult mitochondrial disease, had significantly increased risks of occurrence of GDM, breathing difficulties and hypertension than a contemporaneous comparison group. Despite being asymptomatic at the time of their pregnancy only half of the women with the m.3243A>G mutation had a normal vaginal delivery and almost half of the remaining pregnancies were delivered by emergency caesarean section. Babies born to mothers with m.3243A>G mutations were born at significantly earlier gestations with correspondingly lower birthweights than both the other mutations and the unaffected comparison groups. More than half of these babies were preterm, probably accounting for the high SCBU admission rates.

### Strengths

The effects of pregnancy on women with mitochondrial disease have not been well‐studied, partly due to its rarity. The UK Mitochondrial Disease Patient Cohort, from which this study was derived, is one of the largest cohorts of living patients with genetically confirmed mitochondrial disease in the world.^7^ We learned from women recruited from this cohort that there are a number of under‐recognised complications of pregnancy and delivery in women with mitochondrial disease, in particular the m.3243A>G mutation, which require clinical vigilance and pathophysiological explanation. One further advantage of this study was having two distinct groups for comparison; the first comprising mothers with pathogenic mitochondrial mutations other than m.3243A>G and the second, a group of women not known to harbour a mitochondrial disease‐causing mutation.

### Limitations

A small number of patients and unaffected women withdrew consent for the study after our age‐matching process. Although these dropouts affected the age differences in the other mutations group, they did not significantly affect the basic demographics of this study, especially the age at which these women had their pregnancies. The other mutations group had older participants, reflecting the later age of onset of disease for some mitochondrial genetic defects. Although this study design was primarily reliant on maternal recall of pregnancy and childbirth, with resultant possibility of recall bias due to increasing age and time since relevant pregnancy, it has been demonstrated by a number of studies that such recollections can be clear and accurate.^12^, ^13^ Other drawbacks of a retrospective questionnaire included the presence of self‐selection bias among volunteers who might either over‐report or under‐report their unpleasant experiences. Our patient‐based questionnaire study also lacked the release of hospital and/or midwifery records to confirm the reported experiences of these women. Participants might interpret certain medical terminology dissimilarly from healthcare professionals even though we clarified these via telephone or face‐to‐face interviews. Finally, there could also be confounding factors, such as the changing trends of pregnancies and deliveries over time. Most of the pregnancies and deliveries in this study occurred in the 1990s.

### Interpretation

Among the pregnancy‐related complications in this study, reports of diabetes during pregnancies were significantly higher for mothers with m.3243A>G than in the other two study groups. The population‐prevalence of GDM was widely estimated at between 1 and 3%^14^ before the revised WHO criteria in 2010, which is consistent with the prevalence in women in the unaffected comparison group (2.9%). This emphasises the relatively high rates of GDM in m.3243A>G (16.1%). Diabetes mellitus, which is a recognised phenotype in 38% of m.3243A>G patients,^15^ could account for their poor glycaemic control during pregnancy. Our findings contrast with a study of pregnant women with m.3243A>G mutation in the Netherlands that reported a prevalence of GDM of only 11%,^10^ although this is still higher than in the general pregnant population. Differences in the definition of obstetric complications and different practices as well as varying levels of heteroplasmy might limit any direct comparisons.

Another pregnancy‐related complication observed to be increased was hypertension during pregnancy. More than a third of the pregnancies in women with m.3243A>G had reported high blood pressure compared with those in the comparison group (11.5%) and to international comparative studies of 3.6–9.1%.^16^ High rates of this complication could be attributed to the discrepancy in the interpretation of high blood pressure experienced by or conveyed to patients and the precise technical definition of pregnancy‐related hypertension among clinicians. Given the reliance on precise clinical parameters for a diagnosis of pre‐eclampsia and the limited public understanding of the term we elected not to pursue questions related to this specific diagnosis in the questionnaire. Contrary to diabetes, hypertension is not a known manifestation of m.3243A>G mutations. Placental function is likely to be a key determinant, particularly in relation to the development of hypertension and preterm deliveries. Proteomic alterations relevant to the mitochondrial respiratory chain function and fatty acid oxidation have been reported previously in placentas from women with pre‐eclampsia, where morphological abnormalities of mitochondria were also noted.^2^, ^17^ Recently, impaired mitochondrial function has been demonstrated to play an important role in the development of hypertension and reduced fetal weight in a rat model of pre‐eclampsia.^18^

At the time of delivery, half of the 62 births to mothers with the m.3243A>G mutation required obstetric interventions (instrumental or caesarean section). The rate of interventional delivery in this cohort suggests that the m.3243A>G mutation might be influential in determining the response to metabolic stress associated with the onset of labour. Without the medical records, it was not possible to determine the exact indications for obstetric interventions. As this study collected information on deliveries in the 1990s from the participant, the rates of elective and emergency caesarean sections among m.3243A>G deliveries were unusually high (30.7%) in an era when the rates of these interventions in the UK ranged from 12% in 1990 to 20% in 2000.^19^, ^20^, ^21^ From our limited retrospective assessment, it was difficult to know if the complications observed in pregnancy and emergency caesarean sections were mostly determined by fetal or maternal factors but this could be pursued in prospective studies. Another observation in this study was that there were more preterm babies (<37 weeks) born to mothers with this m.3243A>G mutation even after accounting for multiple births. As a result, they were also more likely to require admission to SCBU. This high rate of prematurity appears to be specific to women with the m.3243A>G mutation and was not observed in women with other pathogenic mitochondrial mutations. Our rate of preterm delivery (53.2%) in these women was much higher than that in the Netherlands study (11%).^10^

Although the pathophysiological process may remain undetermined at present, it is clear from our current study that women harbouring m.3243A>G are at much greater risk of complicated pregnancies and deliveries than those with other forms of mitochondrial disease, who seem to incur very little, if any, additional risk. Our findings have important implications for the obstetric management of pregnancy and delivery in women with the m.3243A>G mutations, even though they might not have any mitochondrial disease manifestation at the time of pregnancy. Women of reproductive age who are discovered to be carriers of the m.3243A>G mutation should be made aware of the increased risks in pregnancy and delivery. We feel this is pertinent given the increasing range of reproductive options (prenatal genetic testing, pre‐implantation genetic diagnosis and mitochondrial donation) that are already available to women who harbour pathogenic mutations associated with mitochondrial disease.^22^, ^23^ Although the novel mitochondrial donation technique might be considered an appropriate reproductive treatment option for some women harbouring the m.3243A>G mutation, it remains unclear if women with the m.3243A>G mutations but pregnant with a fetus with minimal (<2%) mutation load will experience similar pregnancy‐related complications to those reported in this study. Close clinical scrutiny of these mitochondrial donation pregnancies may help to answer the question of whether these complications are a result of fetal or maternal factors.

## Conclusions

Based on our study, we suggest that women with mitochondrial disease, especially those due to the m.3243A>G mutations, are more likely to experience pregnancy‐related problems such as hypertension, GDM, breathing difficulties, emergency caesarean section and preterm birth. Not only will these findings be useful to clinicians in the counselling of expectant women with mitochondrial disease, but they form the basis for future pathophysiological studies into the underlying mechanism of mitochondrial disease in pregnancy.

### Disclosure of interests

All the authors are responsible for recognising and disclosing any conflicts of interests that could be perceived to bias their work, acknowledging all financial support and any other personal connections. RM reports grants from Wellcome, Medical Research Council, Lily Foundation and Ryan Stanford Appeal. AL reports Wellcome Trust 203105/Z/16/Z. There are no conflicts of interest that other authors should disclose having read the statement above. Completed disclosure of interest forms are available to view online as supporting information.

### Contribution to authorship

CF, RT, GG, HD, DT and RM conceived and designed this study. CF, VN and AB interviewed participants and collected clinical data. CF, EF, AL and RM analysed the data with relevant statistics and provided discussion points in the study. CF, EF, AB and JM contributed the necessary materials for data collection, storage and analysis. ABl provided statistical review and generalised linear modelling. JS reviewed and provided crucial interpretation of study results. All authors contributed to the writing of this paper but CF, AL and RM edited the final version. RM is the corresponding author.

### Details of ethics approval

Institutional ethical approval was obtained from Sunderland NRES Committee (Ref 11/NE/0028) on 17 May 2011 and the study complied with the Declaration of Helsinki.

### Funding

This work is supported by the Wellcome Centre for Mitochondrial Research (203105/Z/16/Z), the Medical Research Council (MRC) Centre for Translational Research in Neuromuscular Disease, the Mitochondrial Disease Patient Cohort (UK) (G0800674), the UK NIHR Biomedical Research Centre for Ageing, Newcastle upon Tyne Foundation Hospitals NHS Trust and the UK NHS Highly Specialised Service for Rare Mitochondrial Disorders of Adults and Children.
